# Software for Storage and Management of Microclimatic Data for Preventive Conservation of Cultural Heritage

**DOI:** 10.3390/s130302700

**Published:** 2013-02-27

**Authors:** Ángel Fernández-Navajas, Paloma Merello, Pedro Beltrán, Fernando-Juan García-Diego

**Affiliations:** 1 Departamento de Física Aplicada, Universitat Politècnica de València, Av. de los Naranjos s/n. 46022 Valencia, Spain; E-Mails: afnavajas@fis.upv.es (A.F.N.); palomamerello@outlook.com (P.M.); pbeltran@fis.upv.es (P.B.); 2 Instituto Valenciano de Conservación y Restauración de Bienes Culturales (IVC+R), Complejo Socio-Educativo de Penyeta Roja s/n. 12080 Castellón, Spain; 3 Centro de Tecnologías Físicas, Unidad Asociada ICMM-CSIC/UPV, Universitat Politècnica de València, Av. de los Naranjos s/n. 46022 Valencia, Spain

**Keywords:** relative humidity and temperature sensors, software, database management, mean trajectories, cultural heritage

## Abstract

Cultural Heritage preventive conservation requires the monitoring of the parameters involved in the process of deterioration of artworks. Thus, both long-term monitoring of the environmental parameters as well as further analysis of the recorded data are necessary. The long-term monitoring at frequencies higher than 1 data point/day generates large volumes of data that are difficult to store, manage and analyze. This paper presents software which uses a free open source database engine that allows managing and interacting with huge amounts of data from environmental monitoring of cultural heritage sites. It is of simple operation and offers multiple capabilities, such as detection of anomalous data, inquiries, graph plotting and mean trajectories. It is also possible to export the data to a spreadsheet for analyses with more advanced statistical methods (principal component analysis, ANOVA, linear regression, *etc.*). This paper also deals with a practical application developed for the Renaissance frescoes of the Cathedral of Valencia. The results suggest infiltration of rainwater in the vault and weekly relative humidity changes related with the religious service schedules.

## 1. Introduction

The development of specific software for research purposes, especially in sensor applications, is of high importance in order to adapt to the particular case study and come to relevant conclusions about the recorded data with the maximum possible efficiency [1]. Today, the preventive conservation of the artistic legacy is a priority. Preventive conservation is defined as a working method and a combination of techniques which help in determining and controlling the deterioration process of cultural heritage before it occurs [2].

The practical application of a preventive conservation plan involves the study and control of the risks of deterioration. The objects are influenced by their environment, in terms of stress caused by physical agents such as temperature, humidity, radiation, and chemical agents (e.g., CO_2_, SO_2_, O_3_, mineral salts, *etc.*), and creating sometimes harmful conditions for preservation [3–6]. Specifically, both abrupt changes and not recommended values of temperature and relative humidity (RH) [7], can cause serious damage to the structure of objects, such as non-isotropic material deformation or detachment in materials of several layers. In hygroscopic materials, such as wood panels, which are the mainstay of many artworks, mechanical changes and deformations can occur [8]. In the case of frescoes, moisture and soluble salts are the very common causes of degradation; therefore an early detection of inadequate values of these physical parameters is essential to avoid this kind of damage [9].

It follows that, the study of climatic conditions surrounding the artwork is essential to prevent deterioration [10–13] and to identify eventual consequences of corrective measures. Lately, there is a growing interest in monitoring the climatic parameters in cultural heritage [14–20]. The environmental monitoring is performed by a regular data collection, whose frequencies typically range between one datum every hour (1 datum/hour) or every day (1 datum/day) [14,21]. Data recorded with very low time frequencies (e.g., 1 datum/day) is easy to handle, but valuable information may be lost.

Technological changes and the emergence of faster and cheaper microprocessors have opened the possibility to store and work with larger amounts of data, with higher recording frequencies than a datum per hour [18,19,22,23]. It is possible to take a datum every minute without problem in the current systems of data collection and analysis [15,17]. However, if an accurate grid of the cultural heritage site is monitored, the amount of data recorded increases rapidly, leading to the need to store and manage them properly. Within a few months of monitoring, the amount of recorded data can easily be a million data items [17], making it impractical to use standard business software (MSOffice or OpenOffice). Therefore, it is imperative to develop a database that satisfies requirements of storing large amounts of data, reliability and low-cost compatible with current limited research budgets.

The aim of this paper is to present software specifically developed to store and manage databases (DB) with large storage capacity for environmental monitoring. This paper also presents the application of the DB to the case of the Renaissance frescoes of the Cathedral of Valencia (Spain). For 350 years the frescoes remained in stable conditions of temperature, humidity and lighting, and covered by a Baroque vault. In 2004, the vault was removed to restore the frescoes and they were left uncovered with the intention to be viewed. Furthermore, the outer cover over the vault was reconstructed and waterproofed by an asphaltic roofing sheet which, however, was not tested for tightness and cannot guarantee an absolute impermeability [15,17].

To ensure that there is no water infiltration and that the new environmental conditions do not result in a risk for the long-term conservation, it was decided to implement a monitoring system comprised of temperature and RH sensors installed during the restoration process. The aim of monitoring is double. On one hand, increases of moisture in specific parts of the vault may indicate water infiltration through the roof, which would require corrective measures similar to those specified in [24]. On the other hand, detect diurnal or seasonal excessive thermohygrometric variations to propose corrective measures. In 2006, several salt efflorescence problems were detected in some areas of the frescoes. The monitoring system and management software of the DB play a key role in studying the causes of the salt efflorescences [17].

## 2. Materials and Methods

### 2.1. Software Resources

The software is developed with Delphi [25] using the Object Pascal language and Firebird [26] as database engine. Firebird and Delphi are both developed by the same company (Borland, currently a subsidiary of Micro Focus), favoring its compatibility compared to other open source softwares, like PostgreSQL or MySQL.

**Figure 1.**
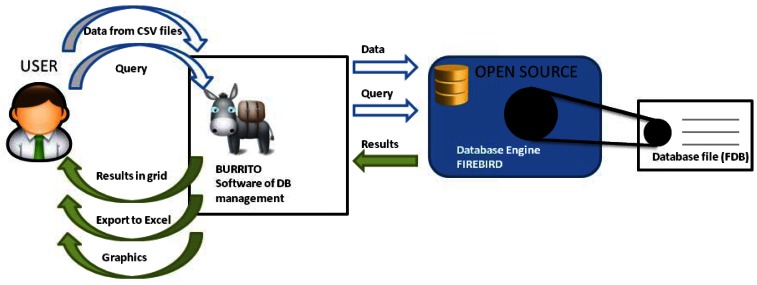
Dynamic behavior between the software, the database engine and the FDB file.

Firebird is open source software; therefore there is no need to pay a license fee for its download, use or the development of commercial programs (Figure 1). It works on MS Windows systems (XP, Vista and Windows 7) in 32 and 64 bit versions. It will possibly work on Linux with a Windows emulator, but this has not been tested. It does not consume excessive system resources, and works perfectly well on a PC equipped with Windows XP and 500 MB of RAM. The DB is a single *.FDB file that can store millions of data points. Currently we manage a DB of over 37 million records and a size of 10.5 GB.

### 2.2. Monitoring System

The monitoring system used in the vault of the Cathedral of Valencia was developed by the research team. The system consists of a microprocessor and a simple wiring system which records data from 32 RH sensors and 32 temperature sensors, placed at different points on the vault and on the surface of the paintings [17]. A frequency of a datum every minute for each sensor is used, generating a volume of more than 2.7 million data *per* month. RH sensors are formed by an HIH-4000 integrated circuit (Honeywell International, Inc., Morristown, NJ, USA) [27] with an accuracy of ±3.5% RH. The temperature sensors are formed by a DS2438 integrated circuit (Maxim Integrated Products, Inc., Sunnyvale, CA, USA) [28] with an accuracy of ±2 °C.

In this work we analyze the data recorded within the period 01/01/2008 to 31/01/2012 (a total of over 37 million records). Also pluviometry data obtained from the National Meteorological Agency [29] is included, for the indicated dates, with a frequency of a datum *per* day.

## 3. Software Description

The software was named Burrito (donkey in Spanish). It is a graphical interface (GUI) between the user and the engine that drives the DB which stores the information recorded by the monitoring system. The main features and functions of the software are described below.

### 3.1. Data-Logger Database File

The software reads the data recorded by the monitoring system in text format, with CSV extensions (comma separated values). These files are quite common in commercial data-loggers. The file must comply with the following template:

YEAR;MONTH;DAY;HOUR;MINUTE;SENSOR1;SENSOR2;SENSOR3…
2011;11;1;0;0;58,96;60,41;56,13
2011;11;1;0;1;59,09;60,22;56,04
2011;11;1;0;2;59,04;60,31;56,2
…

If we see it in table form (Table 1):

It is not possible to obtain a double-digit accuracy for relative humidity and temperature with the current sensors. For the aim of this paper we used the resolution of our data-logger to show that this software can be adapted to situations where different physical parameters are involved, in which cases it is possible that a double-digit accuracy will be necessary.

The first five columns characterize each row, with variables of date and time (year, month, day, hour and minute), and the remaining columns correspond to the sensors (short name) and their data. The sensors can be of different type (RH, temperature, *etc.*) but it is important that the sensor name match its short name in the DB (see Figure 4) for the software to automatically assign the data to the correct sensor. The software is able to incorporate data from a file, regardless of the number of columns and rows it has.

### 3.2. Getting Started

**Figure 2.**
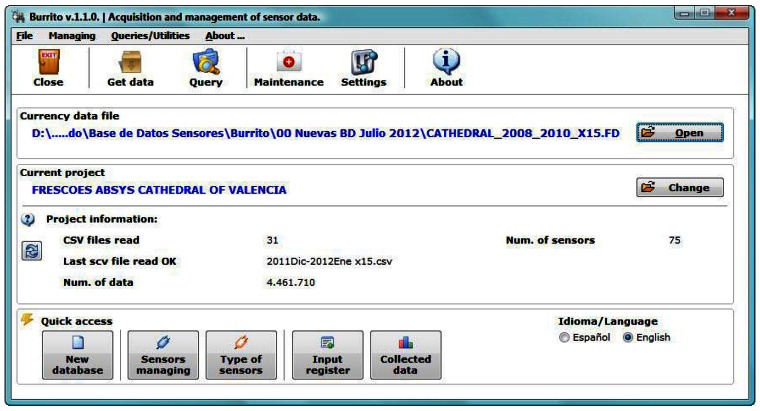
Start-up screen.

Immediately after launching the software the start-up screen appears (Figure 2).

From this screen the software allows quick access to frequently used functions, such as open a DB, add data, query, check the input register, *etc.* From there it is possible to access almost all functions; the additional options are in an attached menu.

The data file from the data loggers must be in comma separated value (CSV) format. On the start-up screen, some details of the DB are shown, such as read CSV data files. It is also possible to manage the language in which the program will be handled (Spanish/English).

The first step to store data recorded by a monitoring system is to create the database file, which will be unique and will have Firebird DataBase (FDB) extension. It is a single file, making its transport, backup, *etc.*, easier. The button “New Database” from the “Quick access” panel (Figure 2) creates a new DB. The file must be named and then will be created an empty FDB file ready to save information. The software allows a single DB to store data from different projects, although this is only recommended in case there is few data points per project. Otherwise, a separate FDB file should be created for each project.

### 3.3. Defining Type of Sensors in the DB

The FDB file is able to store information of any physical parameter such as temperature, RH, lumens, *etc.*, on condition of data have the date and time it was recorded, and their range is 8 bytes.

Given the wide range of physical parameters, before filling the DB, for each physical parameter a different type of sensor must be defined (via the button “Type of sensors” from the “Quick access” panel in the start-up screen, Figure 2). In an empty DB appears a default data type: “Indefinite”. This type of sensor must not be used because it is only for software internal use.

**Figure 3.**
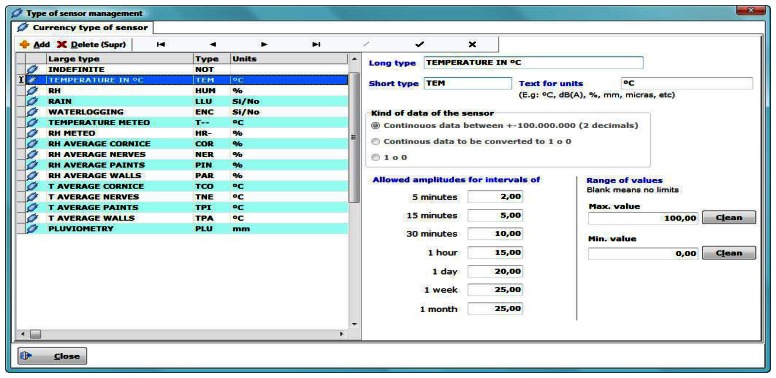
Type of sensor management window.

Inside the sensor type manager (Figure 3) each sensor is defined by its name and abbreviated units, value range and maximum allowable amplitudes for outlier detection. These errors occur due to faults in the communication between the sensor and the microcontroller. Therefore, they do not imply a limitation of the software. Is important to fill this information carefully because the program uses them later to define filters and show useful information.

The program automatically numbers each sensor of each type by its abbreviated name (e.g., HUM001, HUM002, …, HUMn, TEM001, TEM002, …, TEMn etc, for n = 1,2,..999).

The software, when recording new data, filters the values outside the allowed range and does not include them in the DB or marks them as incorrect. The definition of “Allowed amplitudes” (Figure 3), is defined for fast detection of anomalous data. For example, for a maximum allowed amplitude of 10 units for the interval of one hour (hourly averaged data), if the difference between the minimum and the maximum value is greater than 10, the software will display the data in red, indicating a possible error in the recorded data or unusual circumstances in the time interval.

### 3.4. Including Sensors in the DB

**Figure 4.**
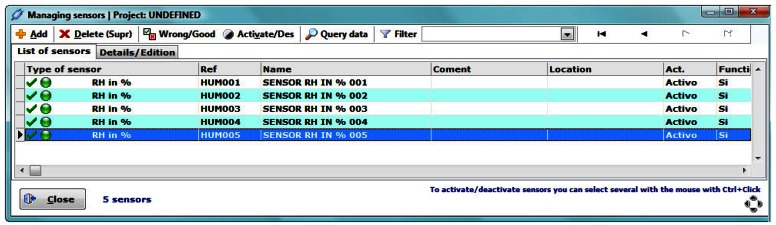
Sensors management window.

After defining the possible types of sensors, is necessary to indicate the number of sensors and their type (from the already defined categories). This can be performed via the button “Sensors managing” (Figure 4), from the “Quick access” panel in the start-up screen (Figure 2).

In the sensor management window (Figure 4) several operations on sensors may be performed, such as mark their performance as good or bad, enable/disable (note that it is not possible to add data to a disabled sensor), check how many data points have been recorded, remove a sensor (only if it does not have recorded data), *etc.*

On the other hand, the CSV file (in correct format) will be loaded into DB in the start-up screen (shortcut F2). The software reads the data from the CSV file, indexes and incorporates them into the DB, informing about when the process ends. At the end of the reading, a report with the errors detected and duplicate data found (data previously included for the same sensor and date) is shown.

The software allows a preview of the data and provides options to configure corrective measures if an anomalous datum is detected (with date/time missing, out of range or out of the maximum allowed amplitudes).

**Figure 5.**
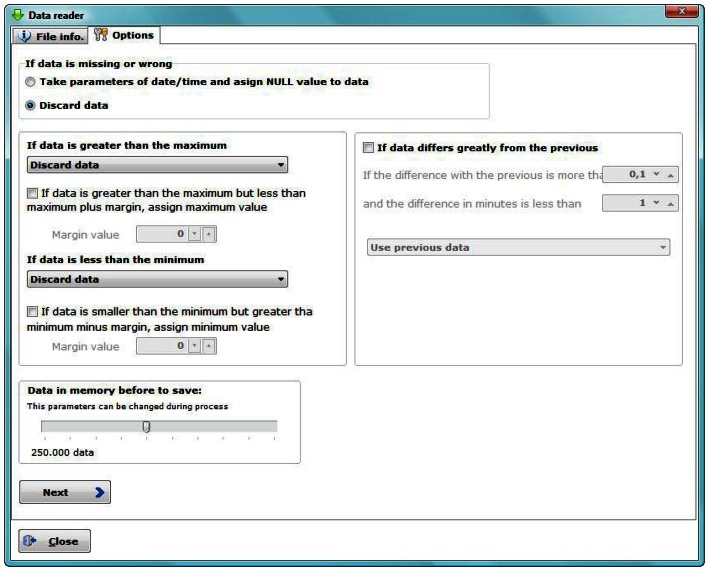
Data reader window.

Thus, it is possible to perform filtering and elimination of erroneous data on the data collected by columns view. In our experience, approximately 2% of the data have some type of failure. After studying in detail each anomalous data, three possible actions could be taken (Figure 5): Delete, mark as erroneous or edit its value (replacing the value by the immediately before/after correct one). Information about anomalous data is stored in a LOG extension file, saved in the same directory as the CSV input data file, for future consults.

### 3.5. Input Register

**Figure 6.**
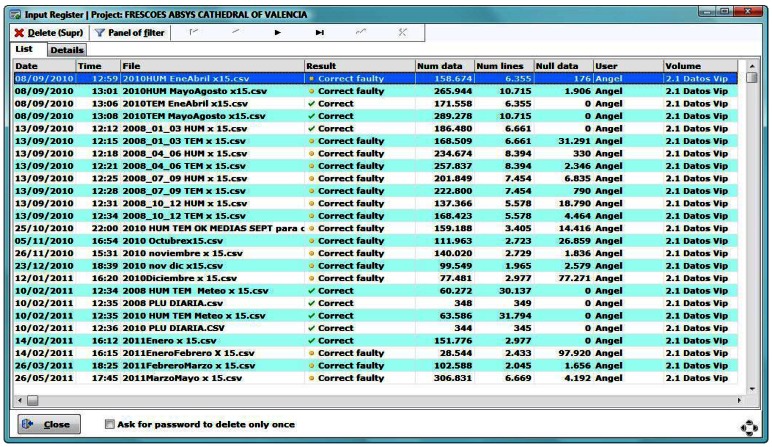
Input register window.

The software keeps a complete register of the information recorded in the DB. This is available by clicking on “Input Register” in the start-up screen (Figure 2). The Input Register shows a list (Figure 6) of all read files, specifies if the result of the reading has been correct, the amount of data recorded, the data discarded, quantity of data *per* sensor, the computer used in the reading, *etc.*

In the Input Register it is possible to delete a CSV file entry (delete from the DB all data entered by that file). Thus, if a data register error takes place it can be easily corrected.

### 3.6. Inquiring

**Figure 7.**
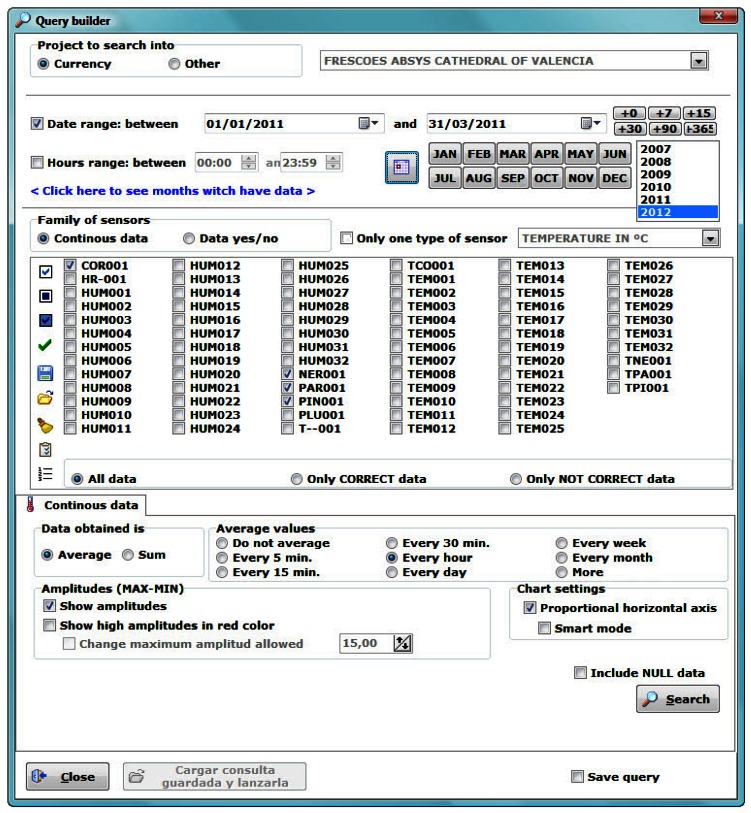
Query builder.

Querying data according to specific criteria is the basic purpose of collecting and storing it. The software allows one to clearly define the queries to be launched to the DB. In the start-up screen (Figure 2, button “Query”) we can set parameters (Figure 7) to determine a specific inquiry (e.g., date range, sensors, averages, *etc.*). The software can provide averaged data (every 5, 15, 30, and 60 minutes, 1 day, 1 week or 1 month). This is very interesting if we work with high frequency monitoring programs which produce huge amounts of data, as it allows showing monitored parameters at shorter intervals without losing relevant information.

For instance, if we select an hourly average, we get a single datum for every sensor and hour, which will be the average of all the data recorded at that hour (e.g., from 00:00 to 00:59), for each day of the considered period. Let us note that for averaging *per* hour it is necessary that the recorded data have a higher sampling frequency than a datum *per* hour, otherwise the software will show only the available data.

**Figure 8.**
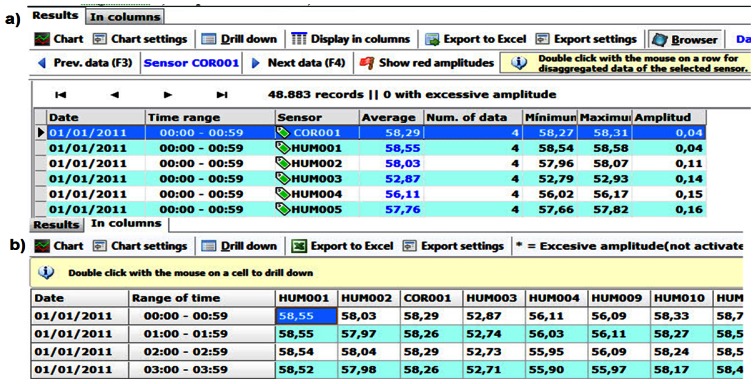
(**a**) Sequential view; (**b**) Column view.

**Figure 9.**
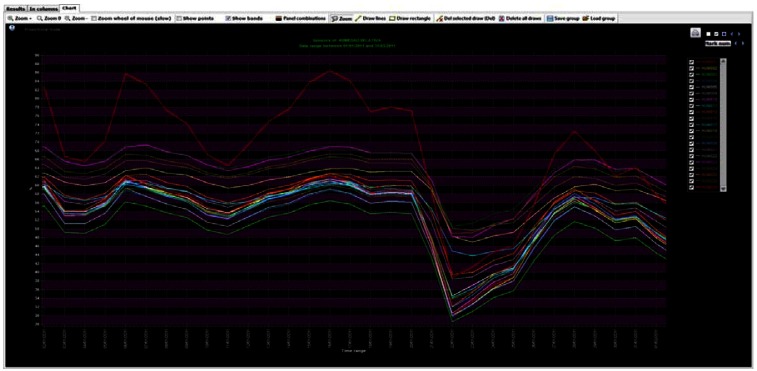
Results plotted on a graph (zoom on the figure for more axis detail).

The query results can be displayed in three different ways: sequentially, as columns and as a graph. The sequence (Figure 8(a)) shows the results in detail, what usually is impractical because it requires checking many rows. The column view (Figure 8(b)) provides less information, although easier to visualize. In the previous display ways, sequential and columns, if you double-click on an item, detailed information of the non-averaged original data is shown. Finally, we can plot a graph (Figure 9), being able to locate every data in the graph and expand its information, in sequential or column view, with a click.

### 3.7. Special Inquiries

The software also allows for special inquiries, as averaging values in the whole period and obtaining mean trajectories. These trajectories are novel and useful in preventive conservation of cultural heritage [18]. Mean trajectories is a plot commonly applied in the control of batch chemical processes because it allows an easy identification of deviations with respect to the target trajectory. However, this kind of plot is not frequently used in microclimate monitoring studies of cultural heritage. The chart provides useful information when cycles are clearly marked.

**Figure 10.**
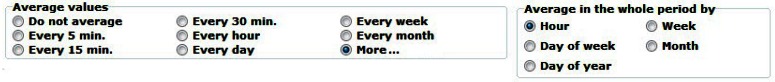
Additional options for averaging.

Average in the whole period allows to obtain the daily mean trajectory, weekly mean trajectory and annual mean trajectory, by means of averaging per hour, day of the week (from 1-Sunday- to 7-Saturday), week of the year (from 1 to 52), day of the year (from 1 to 366) and month (from 1 to 12), for the whole selected period (Figure 10). Afterwards, these mean trajectories can be plotted.

**Figure 11.**
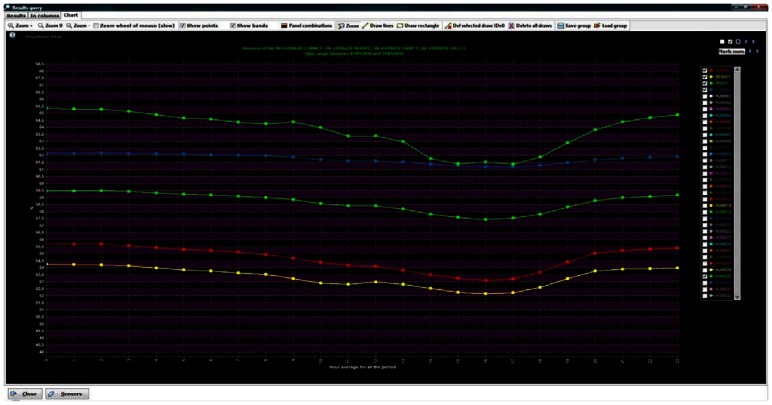
Daily mean trajectories of five sensors (from 01/01/2010 to 03/31/2010). Zoom on the figure for more axis detail.

For instance, if a period from 01/01/2010 to 03/31/2010 is selected, and an averaging *per* hour in the whole period is performed, 24 data are obtained for each selected sensor (the hourly average from 0 hours to 23 hours, Figure 11). The averaged datum for hour = 0 for sensor HUMnnn (e.g.,) is obtained by averaging the data of sensor HUMnnn taken from 0:00 to 0:59, for the whole indicated period.

In the case of the annual mean trajectory (whether averaging *per* day of the year, week of the year or month) data from several years must be recorded, otherwise the annual mean trajectory will coincide with the trajectory of that particular year.

These tools allow us to find patterns in the trajectories of the sensors that respond to hourly or seasonal changes, and propose appropriate preventive measures. Previous studies have pointed out that the flow of visitors rises RH levels above usual levels inside buildings [23,30,31], therefore, changes of pattern associated with public attendance (like Sundays in churches or museums) could be an interesting subject for study by means of the aforementioned mean trajectories.

### 3.8. Data Exportation

The software combines the storage power of a DB and the flexibility of a spreadsheet. It can export inquiry results into a spreadsheet where statistical analysis can be performed as well as elaborate graphical presentations.

After launching a query and getting the results, both from the column view (Figure 8(b)) as from the sequential view (Figure 8(a)), data can be exported to a spreadsheet. The sequential view allows choosing the form of export (sequential/columns). Notice that export as columns is usually more useful.

One can also export the hourly or daily variation (absolute difference of two consecutive values) in case of averaging per hour or per day. It can be safely said that some cultural heritage conservation standards [7,32] recommend values for periodic variations (daily and hourly) where the environment can be considered stable hence less possibilities of having environmentally-related deterioration problems.

### 3.9. More Functions

In addition to the aforementioned functions, the developed software allows one to do backup and restoration of the DB, plot graphs of data recorded per month and per sensor, DB maintenance, and other features that allow keeping the recorded data under control.

## 4. Case Study. Frescoes of the Cathedral of Valencia

### 4.1. Database and Data Pretreatment

In this case, data recorded by the sensors are written by the microprocessor in CSV format on a USB flash drive connected to the system. Periodically, this flash drive is connected to a computer and, using the software, the data is copied to a DB.

**Figure 12.**
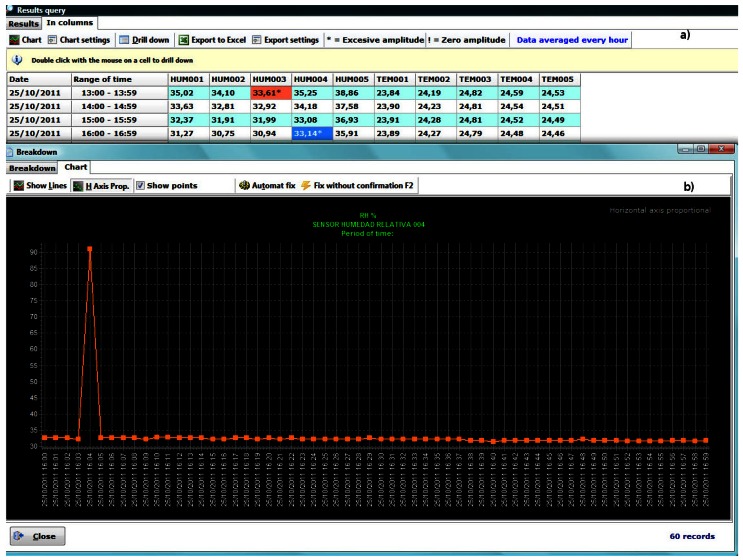
(**a**) Rapid localization of anomalous data in the columns view. (**b**) Localization of outliers in a graph. Zoom on the figure for more axis detail.

A pretreatment was performed on the recorded data. Filtering and elimination of outliers for hourly averaged data by columns view was performed (detecting in red data with amplitudes greater than those allowed for that type of sensor, see Section 3.3). After analyzing the outliers, two different actions were implemented. In those cases where there is more than one consecutive anomalous data (not being able to predict their real value) they were deleted. In cases where the anomalous datum was unique (Figure 12), it was replaced by the immediately previous/subsequent correct value.

### 4.2. Reduced DB for Daily Use

The DB file (FDB extension) currently has over 37 million data points and occupies 10.5 GB on the hard drive. The software can handle this amount of data without problems, but inquiries and backups are slow. To speed up the daily work with the data, a smaller DB was created by averaging data every 15 minutes. This significantly reduces the size of the DB (from over 37 million to 4 million data points) and, consequently, the processing times for inquiries without losing relevant information. This reduced DB is generated from a query in the full DB. An inquiry of data averaged every 15 minutes is performed; results are exported to a spreadsheet and saved in CSV format. This study works with the reduced DB.

### 4.3. Descriptive/Exploratory Analysis

The software allows one to easily and quickly perform exploratory analyses. Concerning the utility of these analyses, the salt efflorescences, detected in 2006 on the walls and frescoes of the vault of the Cathedral, were studied. In order to determine the causes of the efflorescences, the time series of the sensors were analyzed, especially those located in areas where the salt efflorescence appeared.

Studies [15,17] provide information about the vault material and thickness of walls, as well as a sketch showing the location of sensors and their elevations. It should be noted that the city of Valencia has a Mediterranean climate. Furthermore, the vault is located at an elevation of about 15 meters; therefore efflorescence problems are caused by the infiltration of water from the roof but not from the ground level.

**Figure 13.**
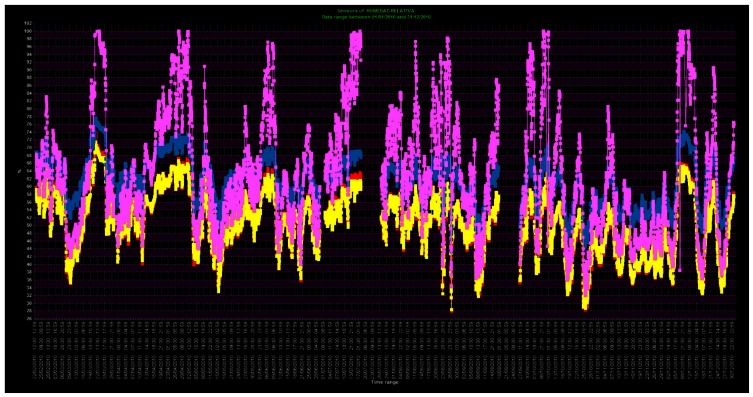
Annual time series of hourly averaged data (2010) of four RH sensors, two of them located in the area with salt efflorescence (SEN3-X blue line and SEN4-X violet line) and two sensors in the area where the frescoes do not show deterioration (SEN1-OK red line and SEN2-OK yellow line). Zoom on the figure for more axis detail.

First, the annual time series for 2010, with data averaged per hour, was plotted (Figure 13). That kind of graph shows an excessive amount of information when large amounts of data are recorded, making it harder to draw useful conclusions.

**Figure 14.**
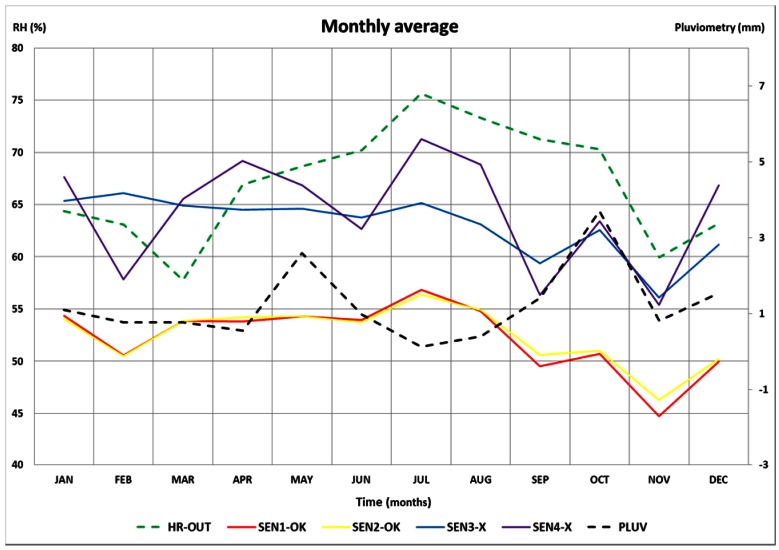
The right vertical axis indicates monthly averaged rainfall data (mm) for PLUV sensor. The left vertical axis indicates mean RH (%) for all other sensors.

In order to detect anomalous behavior in the time series of the sensors, mean annual trajectories were studied. For this purpose, an inquiry of monthly averaged data, for the time period from 1 October 2007 to 31 December 2011, was performed using this software. Six annual mean trajectories of RH were compared. The selected sensors were: two sensors located in the area with salt efflorescences (SEN3-X blue line and SEN4-X violet line), two sensors in the area where the frescoes do not show deterioration (SEN1-OK red line and SEN2-OK yellow line), RH data from outside the Cathedral (RH-OUT), and pluviometry data (PLUV). The results were exported to a MS Excel spreadsheet and plotted as shown in Figure 14, which shows that the signals from the sensors located in areas with salt efflorescences have a higher RH average value than the sensors located in areas without salt efflorescence problems.

Regarding the shape of the trajectories, all sensors emulate the outside RH trajectory but, naturally, moderated by the effect of the walls. However, when the pluviometry exceeds 3 mm per day (October), the sensors in the salt efflorescence area (SEN3-X and SEN4-X) follow the pluviometry pattern, while the sensors SEN1-OK and SEN2-OK continue reflecting the external RH trajectory. As the Cathedral was built of limestone, characterized by a high porosity, therefore the aforementioned results may show the possible infiltration of rainwater into the areas where these sensors are located, helping to determine the most convenient conservation measures.

**Figure 15.**
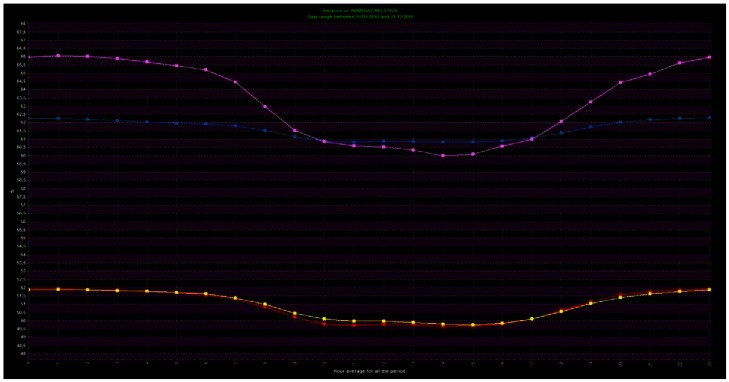
Daily mean trajectories in 2010 (from 1 January 2010 to 31 December 2010) of SEN1-OK (red line), SEN2-OK (yellow line), SEN3-X (blue line) and SEN4-X (violet line). The horizontal axis represents time (hour of the day, from 0 to 23) and the vertical axis represents the averaged RH (%). Zoom on the figure for more axis detail.

The daily mean trajectories of the aforementioned sensors (Figure 15) were also plotted. Figure 15 condenses the common daily pattern in a single graph (different from Figure 13). This figure detects the difference between the sensors in the area with salt efflorescence problems (SEN3-X and SEN4-X) and the ones on the non-problematic area (SEN1-OK and SEN2-OK). The case study of the Cathedral illustrates the power of the software to perform exploratory analyses and find atypical behaviour patterns in the data, helping to extract useful information.

### 4.4. Advanced Statistical Analysis Applications

As mentioned, this software allows data preparation for export and analysis with other software, for example by applying more complex statistical analyses. In previous studies [15,17] Principal Component Analysis (PCA) was applied, allowing identifying different patterns in the sensors trajectories and relating them to the conservation problems detected in some areas of the frescoes.

The software, with its ability to quickly obtain averaged data, ensured that the PCA results in this particular case did not vary substantially if performed with data taken every minute, data averaged every 15 minutes or averaged per hour [15,17].

Let us note that we refer to averages. The reader may wonder why it is needed to record a large volume of data (with a frequency of 1 datum/minute) if the obtained results when one datum per minute is considered agree with those when analyses are performed with one datum per hour. Notice that the difference is that, when working with hourly averaged data, each datum is obtained as an average of the 60 data (1 datum/minute) available for each hour. The results may differ significantly in certain cases when a data is arbitrarily taken at one particular minute in this interval of one hour. The power and confidence of the statistical analysis performed increases in direct proportion to the sample size used [33,34]. This increases the interest of monitoring with frequencies near 60 data per hour (1 datum/minute) and, therefore, the need to develop software able to manage such volumes of data.

The software also helped with the interpretation of the second component PC2, since in a previous study [15] was established that PC2 discriminates sensors according to the shape of the trajectory. The shape of the trajectory is related to hourly, daily or monthly variations, therefore the physical interpretation of PC2 was determined obtaining and studying hourly, daily and monthly averages by means of this software.

## 5. Conclusions

The developed software (Burrito) is a graphic user interface (GUI) that allows the interaction with a DB specifically created to store huge amounts of data such as those generated from environmental monitoring of cultural heritage. The software is intended to be easy to use and powerful, allowing detection of anomalous data, inquiring, plotting mean trajectories and other features which contribute to simplify the management of large amounts of data generated from a long-term monitoring. The software exports the results to a spreadsheet, allowing for advanced statistical studies as PCA.

This software also allows obtaining different averages, which, together with a large amount of data, would give rise a future studies to determine the optimal frequency of monitoring. It presents as a novelty the representation of mean trajectories. The graph provides useful information when cycles are clearly marked because it allows an easy identification of periodic deviations with respect to the target trajectory. Mean trajectories are a type of plot commonly applied in the control of batch chemical processes. However, this kind of plot is not frequently used in microclimate monitoring studies of cultural heritage [18].

Burrito is a low-cost and affordable software because it uses free open source software as database engine; it also runs smoothly on older computers with Windows XP as operating system. This software was specifically designed to manage data generated from long-term monitoring of cultural heritage. However, it could be useful in other scientific fields where obtaining relevant information from data recorded by sensors is necessary, such as agricultural studies where agricultural productivity is related with different physical parameters (RH, temperature, sunshine, rainfall) or solar energy studies related to insolation.

## Figures and Tables

**Table 1.** Data file structure.

**YEAR**	**MONTH**	**DAY**	**HOUR**	**MINUTE**	**SENSOR1**	**SENSOR2**	**SENSOR3**	**…**
2011	11	1	0	0	58,96	60,41	56,13	
2011	11	1	0	1	59,09	60,22	56,04	
2011	11	1	0	2	59,04	60,31	56,20	
…								
